# Heart‐focused breathing technique and attitude breathing technique effects on heart rate variability in young healthy subjects

**DOI:** 10.14814/phy2.70589

**Published:** 2025-11-04

**Authors:** Ilinca Savulescu‐Fiedler, Sandica Bucurica, Ioana Toader, Constantin Pistol, Ionela Maniu

**Affiliations:** ^1^ Department of Internal Medicine “Carol Davila” University of Medicine and Pharmacy Bucharest Romania; ^2^ Department of Internal Medicine Coltea Clinical Hospital Bucharest Romania; ^3^ Department of Gastroenterology “Carol Davila” University of Medicine and Pharmacy Bucharest Romania; ^4^ Department of Gastroenterology University Emergency Central Military Hospital “Dr. Carol Davila” Bucharest Romania; ^5^ Physics Department University of Bucharest Bucharest Romania; ^6^ Department of Mathematics and Informatics, Faculty of Sciences Lucian Blaga University Sibiu Sibiu Romania; ^7^ Research Team Pediatric Clinical Hospital Sibiu Sibiu Romania

**Keywords:** anxiety, attitude breathing technique, heart‐focused breathing technique, HRV, normalized coherence

## Abstract

Heart rate variability (HRV) is controlled mainly by the autonomic nervous system (ANS). The aim of our prospective study was to investigate whether respiratory control influences changes in ANS activity, as reflected by HRV parameters. The analyzed parameters were compared in different sessions and by anxiety or depression score. The root mean square of the mean squared differences of successive NN intervals (rMSSD) and the high frequency power (HF) was lower in anxious females. As compared with rest, in sessions that involved respiratory control, the standard deviation of NN intervals (SDNN) and low frequency power (LF) were higher, whereas the ratio between very low frequency (VLF) and total power (TP) had lower values. LF/HF expressed the highest variation in all analyzed groups, increasing in all situations compared to the rest, without differences between sexes. Normalized coherence (NCoh) peaked during heart‐focused breathing and was lowest at rest. NCoh increased during respiratory control in anxious females. The NCoh‐LF/HF correlation was observed in all sessions in subjects without anxiety. Respiratory control improved HRV parameters and cardiorespiratory coherence, with significant differences between sexes and individuals with and without anxiety.

## 1 INTRODUCTION

The autonomic nervous system (ANS) influences heart activity depending on physiological conditions. In resting conditions, vagal tone prevails over the sympathetic tone (Kim et al., 2018). During physiological arousal, both sympathetic and parasympathetic activities are increased, with dominance of the sympathetic activity (Giuliano et al., 2017).

Heart rate variability (HRV) is a parameter that reflects the subtle variations between consecutive heartbeats. HRV is influenced by multiple individual factors, such as age (it decreases with age), sex (during the hormonally active period, HRV at rest has higher values in females), and circadian rhythm (it increases overnight and decreases markedly in the first hours of mornings) (Rodríguez‐Colón et al., 2014). HRV is also influenced by lifestyle, posture, and respiration; physically active people have higher HRV (Fatisson et al., 2016). Environmental factors such as exposure to electromagnetic fields, vibrations, and psychosocial pressure also influence HRV (Aubert et al., 2003; Bernardi et al., 2000; Fatisson et al., 2016; Routledge et al., 2010; Sandercock et al., 2005).

Respiration and heart activity are strongly connected: inspiration causes an increase in heart rate, and exhalation has the opposite effect. The afferent inputs from the heart and breathing influence some brain areas involved in emotion regulation, mainly the prefrontal regions (Mather & Thayer, 2018). The situation in which cardiac and cerebral activities such as awareness, reasoning, and emotions are coordinated defines coherence. The increase in the amplitude of heart rate oscillations promotes functional connectivity between various brain regions, particularly those involved in emotional regulation. Cardiac coherence is observed in various spiritual practices (mantra yoga, recitation of the rosary, and prayer of the heart) (Bernardi et al., 2001).

Coherence is a statistical index that reflects how strongly two ergodic signals are connected through a linear relationship, with nonlinear effects or noise reducing the value. Also termed cross‐coherence, it denotes the synchronization of oscillatory systems, such as breathing and heart rate, when they become aligned and function at a similar frequency (McCraty & Shaffer, 2015).

Heart coherence, also called cardiac coherence or resonance, is assessed through HRV analysis and occurs when the heart rhythm adopts a more regular, sine wave–like pattern at approximately 0.1 Hz (about a 10‐s cycle). A coherent rhythm appears as a harmonic, high‐amplitude, narrow peak in the low frequency (LF) range of the HRV spectrum, without notable peaks in the very low frequency (VLF) or HF ranges. Measurement involves locating the dominant peak between 0.04 Hz and 0.26 Hz, computing the power within a 0.030 Hz window centered on that peak, and comparing it to the total spectral power ((McCraty & Shaffer, 2015).

One study in the HRV biofeedback field, which analyzed 1.8 million biofeedback sessions, showed that the highest coherence scores correlate with sinusoidal oscillations of the heart rate around the frequency of 0.1 Hz, within the frequency range of 0.049–0.01016 (Balaji et al., 2025; Fatisson et al., 2016; McCraty & Shaffer, 2015). The breathing cycle period for a frequency of 0.1016 Hz is approximately 9.84 s (Balaji et al., 2025). Breathing with a frequency of 6 breaths/minute leads to a resonance frequency associated with better ANS activity, better physiological functioning, and improved well‐being (Balaji et al., 2025; Chaitanya et al., 2022; Lehrer & Gevirtz, 2014; McCraty, 2022; McCraty & Childre, 2010; McCraty & Zayas, 2014).

Cardiac coherence (resonance) can be voluntarily achieved by controlling the respiratory rhythm, specifically by slowing it down to a breath every 10 s, which corresponds to a resonance frequency of 0.1 Hz (Lalanza et al., 2023; Sevoz‐Couche & Laborde, 2022).

### 1.1 Objectives

The main objectives are represented by the assessment of HRV in the fields of time and frequency at rest, during exposure to negative images, and self‐controlled breathing through the heart‐focused and attitude‐breathing techniques, and by the analysis of the influence of one particular breathing modulation technique (the attitude breathing technique) on individual response at stressor triggers (negative images). A secondary objective was represented by comparing the parameters analyzed for different sessions and analyzing the parameters according to anxiety or depression scores.

## 2 MATERIALS AND METHODS

This prospective study involved resident doctors of similar age, with similar academic training, and in apparent good health. Resident doctors were chosen due to the emotional demands of their profession, as clinical duties routinely involve situations that require sustained emotional regulation (Lundin et al., 2018). To ensure sample homogeneity, participants had similar professional experience, comparable ages, were in good health, and were not on regular medication. The study's theoretical basis and aims have been explained, and the subjects were included only with their agreement. All participants signed an informed consent form. All participants were evaluated for depression, using the Hospital Anxiety and Depression Scale (HADS) (Balan et al., 2020; Velescu et al., 2024; Wu et al., 2021). The duration of HRV recordings in different circumstances was the same for each study participant. Recordings were done using the emWave Pro sensor produced by the Heart Math company. Recordings have been made in a closed room, in silence, with a temperature of 20–24 degrees, with the singular participation of the person designated for making the recordings. All electronic devices (watches and phones) were removed.

The study participants were informed that 1 h before the recordings, they should not consume alcohol, coffee, nicotine, chocolate, or energy drinks; 30 min before, they should not smoke; and 12 h before the recordings, they should not make any particular physical effort (Catai et al., 2020). The recordings were performed in the morning (between 8:30 and 11:00). The method of obtaining cardiac coherence, the heart‐focused breathing technique, as well as the attitude breathing technique, was all explained and demonstrated beforehand. The breathing technique consisted of focusing attention on the heart area. Participants were encouraged to imagine their breath is flowing into and out of their heart, breathing a little slower and deeper than usual. Breathing should be balanced—5‐s breath in and 5‐s breath out. The breathing rhythm—inhale and exhale—was assisted by the trainer raising and lowering their arm. Before the study began, HADS was administered by the same trained person. Blinding procedures were applied.

The recording preparation period was represented by 10 minutes of relaxation while sitting in a comfortable chair with a backrest and arms.

The study was conducted between May 28, 2024, and October 1, 2024, with ethics approval from the Ethics Committee of Coltea Clinical Hospital (No. 6006/10.04.2024).

Written informed consent was obtained from all subjects involved.

### 2.1 Recordings, after the preparation period

Recording at rest, in a sitting position, without any stimulus, uncontrolled breathing rhythm—recording duration 5 min (session K);

Break 5 min;

Recording during the presentation of images with negative content (25 images, presented in 5 min—12 s/image) (session L);

Break 5 min;

Recording with respiratory rhythm control to obtain cardiorespiratory coherence (rhythm 6 breaths/minute, 0.1 Hz) for 5 min (session M). According to Heart Math protocols, this is obtained using the heart‐focused breathing technique (“Focus your attention in the area of the heart. Imagine your breath is flowing in and out of your heart or chest area, breathing a little slower and deeper than usual. Find an easy rhythm that's comfortable.”).

Exposure to images with negative content (the same 25 images from folder 3, for 5 min, in the same rhythm, one image in 12 s) while the subject has control over the breathing rate as well as control over the type of emotion that must replace the potential negative one—attitude breathing technique (session N). This technique respects the Heart Math protocols listed below: (“Step 1. Recognize the feeling or attitude you want to change and identify a replacement attitude. Step 2. Focus your attention on the heart area. Imagine your breath flowing in and out of your heart or chest area, breathing more slowly and deeply than usual. Find a comfortable rhythm. Step 3. Breathe the feeling of the new attitude slowly and casually through your heart area.”).

We used 5‐min short‐term recordings of cardiac electrical activity. The cardiac signal oscillations, converted into intervals or different frequency distributions, are reported numerically (Catai et al., 2020). HRV assessment is done through analysis in the time and spectral domains.

The exposure time of 5 min was established based on previous data indicating that a minimum of 3 min of exposure is necessary to achieve the physiological adjustment required for significant mood changes and emotional responses (Mizumoto et al., 2025). Moreover, this time enabled recording in all frequency bands and time intervals.

The main measurements in the time domain derive from the direct measurement of the NN intervals or the measurement of the differences between the NN intervals. The time and spectral domain metrics represent the analyzed parameters, as they are synthetically presented in Table 1.

**TABLE 1:** Analyzed heart rate variability parameters.

Parameter	ANS—sympathetic and parasympathetic contributions
The standard deviation of the NN intervals (SDNN) (ms)	Sympathetic and parasympathetic contribution
The root mean square of the mean squared differences of successive NN intervals (rMSSD) (ms)	A marker of the parasympathetic nervous system activity
Total power (TP) (ms^2^/Hz)—the sum of the PSD in the range 0–0.4 Hz	Sympathetic and parasympathetic contribution
High frequency power (HF) (ms^2^/Hz)—the sum of the PSD in the range ≥0.15 to <0.4 Hz	A marker of the parasympathetic nervous system activity
Low frequency power (LF) (ms^2^/Hz)—the sum of the PSD in the range ≥0.04 to <0.15 hertz	Reflects sympathetic and parasympathetic activities, but more of the sympathetic activity
Very‐low frequency power (VLF) (ms^2^Hz)—the sum of the PSD in the range ≥0.0033 to <0.04 hertz	Increased in sympathetic nervous system activity
Ultra‐low frequency power (ULF) (ms^2^/Hz)—the sum of the PSD frequencies <0.0033 Hz	It is not clear which is the sympathetic or the parasympathetic nervous system
Normalized LF (nLF/nlf)^a^ (nu)	Reflects sympathetic and parasympathetic activities, but more of the sympathetic activity
Normalized HF (nHF/nhf)^a^ (nu)	A marker of the parasympathetic nervous system activity
LF/HF The ratio of LF power to HF power	The global sympathetic/parasympathetic balance
Normalized coherence (NCoh) (%) The normalized coherence level is determined by measuring the PSD around the largest peak in the coherence range and dividing it by the PSD total power. Normalized coherence ranges from 0 to 100.	It represents a measure of the degree of coherence in the heart rhythm pattern. The coherent heart rhythm is defined as a stable, regular, repeating rhythm resembling a sine wave at a single frequency between 0.032 and 0.26 Hz (2–15 cycles per minute)—the more stable and regular the heart rhythm frequency, the higher the coherence score.

*Note*: SDNN is highly correlated with TP, ULF, VLF, and LF spectral power (Umetani et al., 1998), and rMSSD with HF (Kleiger et al., 2005), rMSSD and HF both appreciate parasympathetic tone, rMSSD being less affected by respiratory movements than HF (Hill & Siebenbrock, 2009; Penttilä et al., 2001). SDNN, TP, LF, and VLF are lower in females than in males; however, HF has higher values in females (Koenig & Thayer, 2016).

Abbreviation: PSD, power spectral density.

^a^ nLF = LF/TP−VLF (Koenig & Thayer, 2016), nlf = LF/HF+LF (Wu et al., 2021); nHF = HF/TP−VLF (Koenig & Thayer, 2016), nhf = HF/HF+LF (Burr, 2007; Heart rate variability, 1996; McCraty et al., 2009; McCraty & Shaffer, 2015; Shaffer et al., 2014; Shaffer & Ginsberg, 2017).

The minimum time required for SDNN, rMSSD, and HR recordings is 1 min (Salahuddin et al., 2007; Shaffer & Ginsberg, 2017).

Recording of all the spectral bands that comprise the TP band is done for 5 min (Salahuddin et al., 2007; Shaffer & Ginsberg, 2017; Umetani et al., 1998).

The coherent ratio is defined by the following equation: CR = peak power/total power below the peak frequency. The coherence score (CS) is a parameter derived from CR used to establish the amount of coherence represented. CS values range from 0 to 8 (Balaji et al., 2025).

### 2.2 Data analysis and statistics

We examined heart rate and heart rate variability (HRV) in the time domain (SDNN and rMSSD) as well as in the frequency domain (TP, HF, LF, and VLF). Relative values were also assessed: normalized (nLF, nHF, LF/HF, HF+LF/TP, HF+LF/VLF, and VLF/TP). Additional parameters included normalized coherence (NCoh), which was also calculated. The normalized components were nLF = LF/(LF + HF) and nHF = HF/(HF + LF) (Burr, 2007). Processing was performed in MATLAB (MathWorks, Natick, MA, USA) and R v4.0.5, and the graphical displays obtained show the variations in depression and anxiety scores according to gender and age.

It was performed a linear regression analysis using the “fitlm” function from Matlab, to model the relationship between a response variable (the collected parameters of interest: FC, SDANN, TP, LF, VLF, ULF, rMSSD, HF, Ncoh, etc.) and one or more predictor variables (Sex and Age, Sex and Depression Score, or Sex and Anxiety Score). Going further, the table resulting from the linear regression analysis was included in a two‐way ANOVA, which provides information about levels of variability within the regression model and forms a basis for tests of significance (Hagen, 1999).

Later, we analyzed sessions from the entire sample, as well as subgroups split by median age (26–28 years), anxiety (0–7 vs. 8–21) score groups, and depression (0–7 vs. 8–21) score groups. For each comparison, *t*‐tests or repeated measures analysis of variance (ANOVA), supplemented by a Bonferroni post hoc test, were performed. Parametric relationships were displayed using Spearman's rank correlation and correlograms.

## 3 RESULTS

Of the 31 participants recruited initially, 30 attended and completed both sessions. Distortive artifacts (e.g., missed or spurious beats) for Heart Rate Variability (HRV) measures were eliminated according to established criteria (Peltola, 2012).

The total number of analyzed subjects (the general lot—GL) was 24, comprising 17 women (F) and 7 men (M), with an average age of 27.04 years (SD = 1.97). The mean score for anxiety was 7.04 (SD = 3.97). The mean depression score was 3.13 (SD = 2.27).

The subjects were grouped according to the anxiety score into two subgroups: the subgroup A 0–7 (anxiety score 0–7) = 15 subjects (7 male and 8 female subjects) and the subgroup 8–21 (anxiety score 8–21) = 9 subjects (no male and nine female participants).

Depending on the depression score, two other subgroups were formed: the subgroup D0–7 (depression score 0–7), comprising 22 subjects (7 males and 15 females), and the subgroup D8–21 (depression score 8–21), comprising two subjects (0 males and 2 females).

The following lot studies were established: the general cohort study (GL), the A0–7 and A8–21 subgroups, and the D0–7 and D8–21 subgroups. The number of subjects with a depression score >8 was too small to be included in the statistical analysis.

### 3.1 The comparative parameters' evolution between sessions

Parameters' evolution is presented in Table 2 (the general study cohort), in Table 3 (subjects without anxiety), and in Table 4 (subjects without depression).

**TABLE 2:** The main HRV parameters evolution in the general study cohort (GL).

GL—(24p)	L vs. K		N vs. K		N vs. L		M vs. K		*p*
	F	M	F	M	F	M	F	M	*η* ^2^
	(*p* value L vs. K)		(*p* value N vs. K)		(*p* value N vs. L)		(*p* value M vs. K)		
TP					+	+			0.168
					0.0491				0.075
LF			+	+	+	+	+	+	
			0.0514*		0.0057*		0.0108*		
VLF+ULF					−	+			
					0.0336^#^				
VLF/TP			−	−	−	−	−	−	0.000
			0.0096*		0.0016*		0.0001*		0.489
VLF+ULF/TP			−	−	−	−	−	−	0.000
			0.0007*		0.0006*		0.0000*		0.513
HF+LF/TP			+	+	+	+	+	+	0.000
			0.0007*		0.0007*		0.0000*		0.512
HF+LF/VLF+ULF			+	+	+	+	+	+	0.001
			0.0029*		0.0058*		0.0013*		0.297
HF+LF/VLF			+	+	+	+	+	+	0.005
			0.0139*		0.0142*		0.0041*		0.239
nLF	++	+	+	+			+	+	0.000
	0.0273^#^		0.0100*				0.0000*		0.326
nlf			+	+			+	+	0.000
			0.0349*				0.0001*		0.285
nHF							−	−	0.000
							0.0004*		0.258
nhf	−	−	−	−	−	−	−	−	0.000
	0.0349*		0.0349*		0.0405*		0.0001*		0.285
LF/HF	−	+	+	+	+	+	+	+	0.002
	0.0019^#^		0.0108*		0.0298*		0.0012*		0.294
NCoh			+	+	+	+	+	+	0.000
			0.0000*		0.0000*		0.0000*		0.705

*Note*: +, higher; −, lower; #, differences between sexes; *, differences between sessions; ++, a higher increase; General lot (GL)—24 patients, Basal (K); negative images (L); heart‐focused breathing (M); attitude breathing during exposure to negative images (N); nLF = LF/TP−VLF, nlf = LF/HF + LF, nHF = HF/TP−VLF, nhf = HF/HF + LF; *p* and *η* ^2^ are the significance and the effect size measures (partial eta‐squared *η* ^2^) of the repeated measures analysis of variance ANOVA test.

Abbreviations: HF, high frequency; LF, low frequency; NCoh, normalized coherence; rMSSD, root mean square of the mean squared differences of successive NN intervals; SDNN, standard deviation of NN intervals; TP, total power; ULF, ultra‐low frequency; VLF, very low frequency.

**TABLE 3:** Comparison between HRV parameters in subjects without anxiety.

A0–7—(15p)	L vs. K		N vs. K		N vs. L		M vs. K		*p*
	F	M	F	M	F	M	F	M	*η* ^2^
	(*p* value L vs. K)		(*p* value N vs. K)		(*p* value N vs. L)		(*p* value M vs. K)		
SDNN			+	+	+	+	+	+	0.002
			0.0265*		0.0281*		0.0162*		0.369
TP					+	+			0.144
					0.0563*				0.131
LF					+	+	+	+	0.019
					0.0233*		0.0082		0.249
VLF/TP					−	−	−	−−	0.000
					0.0161*		0.0000*		0.613
							0.0377^#^		
VLF+ULF/TP			−	−	−	−	−	−	0.000
			0.0019*		0.0086*		0.0000*		0.628
HF+LF/TP			+	+	+	+	+	+	0.000
			0.0119*		0.0091*		0.0000*		0.627
HF+LF/VLF+ULF			+	+	+	+	+	+	0.023
			0.0094*		0.0061*		0.0147*		0.310
HF+LF/VLF			+	+	+	+	+	+	0.047
			0.0181*		0.0057*		0.0368*		0.248
nLF	++	+					+	+	0.001
	0.0273^#^						0.0001*		0.311
nlf			+	+			+	+	0.003
			0.0349*				0.0010*		0.279
nHF							−	−	0.005
							0.0024*		0.260
nhf							−	−	0.003
							0.0010*		0.279
LF/HF	−	+					+	+	0.013
	0.0019^#^						0.0037*		0.325
NCoh			+	+	+	+	+	+	0.000
			0.0001*		0.0002*		0.0000*		0.672

*Note*: +, higher; −, lower; #, differences between sexes; *, differences between sessions; −−, a greater decrease; ++, a higher increase; subjects with anxiety scores <7 (A0–7)—15 patients; Basal (K); negative images (L); heart‐focused breathing (M); attitude breathing during exposure to negative images (N); nLF = LF/TP‐VLF, nlf = LF/HF + LF, nHF = HF/TP‐VLF, nhf = HF/HF + LF; *p* and *η* ^2^ are the significance and the effect size measures (partial eta‐squared *η* ^2^) of the repeated measures analysis of variance ANOVA test.

Abbreviations: HF, high frequency; LF, low frequency; NCoh, normalized coherence; rMSSD, root mean square of the mean squared differences of successive NN intervals; SDNN, standard deviation of NN intervals; TP, total power; ULF, ultra‐low frequency; VLF, very low frequency.

**TABLE 4:** Comparison between HRV parameters in subjects without depression.

D0–7—(22p)	L vs. K		N vs. K		N vs. L		M vs. K		*p*
	F	M	F	M	F	M	F	M	*η* ^2^
	(*p* value L vs. K)		(*p* value N vs. K)		(*p* value N vs. L)		(*p* value M vs. K)		
SDNN					+	+			0.024
					0.0241*				0.167
TP					+	+			0.180
					0.0498*				0.079
LF			+	+	+	+	+	+	0.007
			0.0548*		0.0061*		0.0130*		0.204
VLF					+	+			0.097
					0.0580^#^				0.102
HF+LF/TP			+	++	+	+	+	++	0.000
			0.0006*		0.0003*		0.0000*		0.575
			0.0334^#^				0.0284^#^		
HF+LF/VLF+ULF			+	+	+	+	+	+	0.000
			0.0019*		0.0041*		0.0008*		0.326
HF+LF/VLF			+	+	+	+	+	+	0.000
			0.0111*		0.0117*		0.0131*		0.263
nLF	++	+	+	+			+	+	0.000
	0.0273^#^		0.0132*				0.0000*		0.317
nlf			+	+			+	+	0.000
			0.0279*				0.0001*		0.289
nHF							−	−	0.000
							0.0003*		0.266
nhf			−	−			−	−	0.000
			0.0279*				0.0001*		0.474
LF/HF	++	+	+	+	+	+	+	+	0.001
	0.0495^#^		0.0153*		0.0422*		0.0006*		0.326
NCoh	+	++	+	+	+	+	+	+	0.000
	0.0495*		0.0000*		0.0000*		0.0000*		0.716

*Note*: +, higher; −, lower; #, differences between sexes; *, differences between sessions; ++, a higher increase; subjects without depression (score <7, scale between D0 and D7)–22 patients; basal, (K); negative images (L); heart‐focused breathing (M); attitude breathing during exposure to negative images (N); nLF = LF/TP‐VLF, nlf = LF/HF + LF, nHF = HF/TP‐VLF, nhf = HF/HF + LF; *p* and *η* ^2^ are the significance and the effect size measures (partial eta‐squared *η* ^2^) of the repeated measures analysis of variance ANOVA test.

Abbreviations: HF, high frequency; LF, low frequency; NCoh, normalized coherence; rMSSD, root mean square of the mean squared differences of successive NN intervals; SDNN, standard deviation of NN intervals; TP, total power; ULF, ultra‐low frequency; VLF, very low frequency.

### 3.2 Analysis of HRV parameter with age, anxiety or depression score, and between sessions

No changes in heart rate (HR) were recorded between sessions. Regarding the evolution of time domain parameters, neither SDNN nor rMSSD differed in relation to age, anxiety, or depression scores.

Still, SDNN differed in sessions that involved respiratory intervention (controlled respiratory rhythm or attitude breathing technique) compared to sessions without respiratory control (K, L), only in the A0–7 and D0–7 subgroups. SDNN increased in both sexes, without differences between sexes, in all sessions involving respiratory control (M, N) compared to sessions without respiratory control, observed in subjects without anxiety (N vs. L and M vs. K) or depression (N vs. L).

All values of SDNN in females with depression were lower compared to those without depression, for all K‐N sessions. In females with anxiety, SDNN values in the L session were lower than in the K session (*p* = 0.0402).

The only variation for rMSSD was observed in females with anxiety—rMSSD decreased in M session versus K (*p* = 0.0262). The results for the spectral domain parameters showed only a few variations in terms of absolute values. The TP, LF, and VLF parameters showed no variations with age, anxiety, or depression score. However, TP registered an increase in the session N versus L, in all groups analyzed, without differences between sexes.

For LF, the only variations between sessions, with no differences between sexes, were recorded for the sessions with respiratory control (M and N) versus those without respiratory control (K, L). LF values were higher in the respiratory sessions compared to non‐respiratory sessions in the general study population, in subjects without depression (M vs. K, N vs. K, and N vs. L), and were limited in those without anxiety (M vs. K and N vs. L). LF values in F with depression were lower than LF values for those without depression, for all sessions (K–N).

VLF evolution was different in males and females in sessions M and N. VLF values were higher in male participants in the M session (*p* = 0.0250), with a divergent trend in males and females in the N session (*p* = 0.0495). In females with anxiety, VLF power was lower during the heart‐focused breathing technique session than in the basal session (*p* = 0.0158) and during the attitude breathing technique session compared to the basal session (*p* = 0.0447).

At rest, ULF remains constant with age or sex. In the session using the heart‐focused breathing technique, however, ULF evolved differently in males and females, with higher values in males and a distinct trend compared to VLF.

**FIGURE 1:**
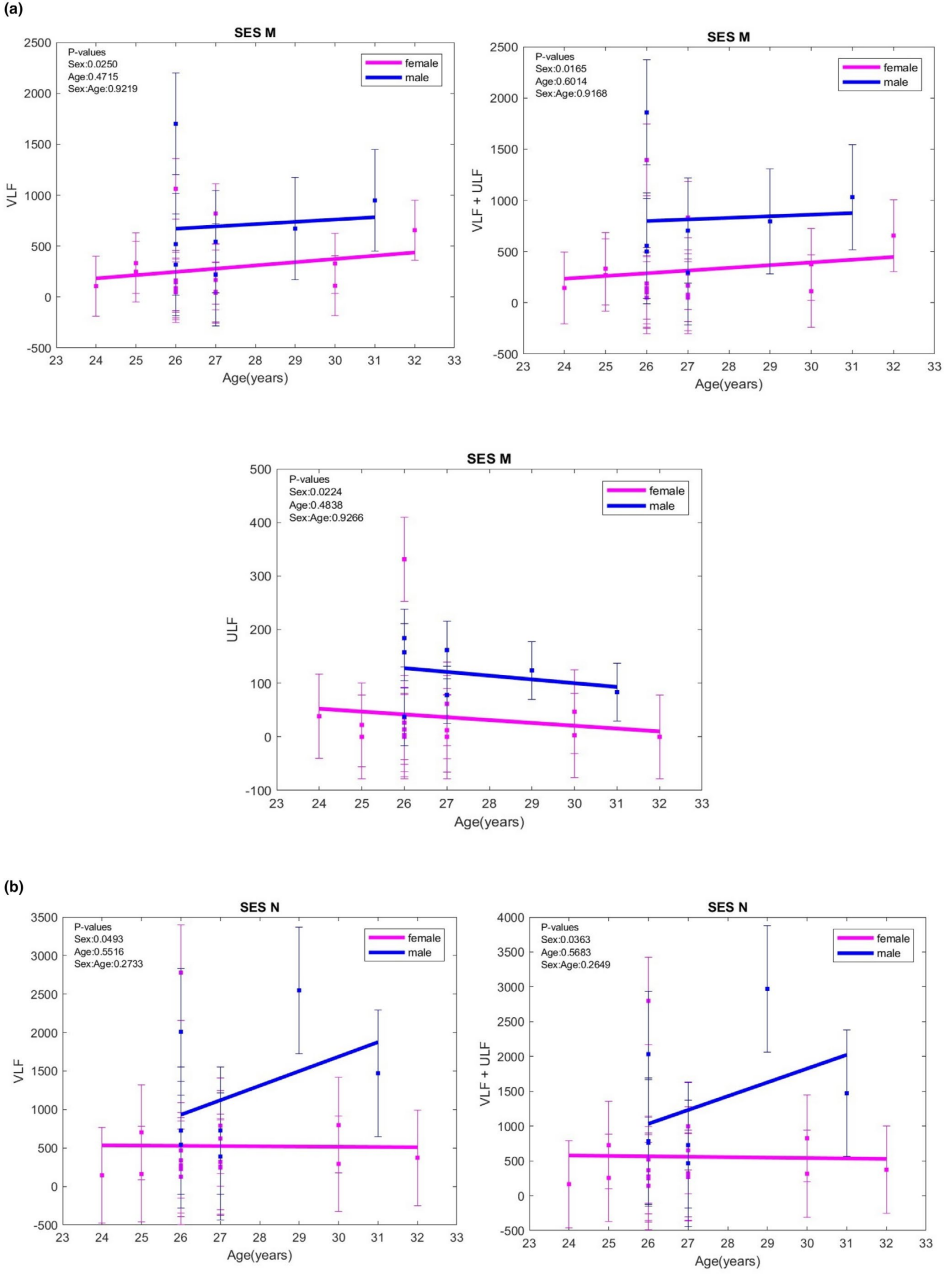
(a) VLF, ULF, and VLF+ULF variations with age in the heart‐focused breathing (M) session; (b) VLF and VLF+ULF variations with age in the attitude breathing (N) session; ULF, ultra‐low frequency; VLF, very low frequency. The error bars represent ± one standard deviation (SD) from the mean value: Basal (K), negative images (L), heart‐focused breathing (M), attitude breathing during exposure to negative images (N).

VLF+ULF evolved similarly to that of VLF for heart‐focused and attitude breathing technique sessions. For the Heart‐focused breathing session, VLF+ULF was higher in males (*p* = 0.0165). For the attitude breathing technique session, the evolution of VLF+ULF was divergent, decreasing in females with age in females and increasing with age in males, with higher values in males (*p* = 0.0363). As for VLF, as well as VLF+ULF, were lower in females with anxiety in the heart‐focused breathing technique than in the basal session (*p* = 0.0099). (Figure 1).

There were no variations of HF between sessions or with age, anxiety, or depression score. For the heart‐focused breathing technique session, HF evolved differently in females, decreasing with age, and increasing with age in males (*p* = 0.0217).

There were no variations of HF with the anxiety score. As for rMSSD, in females with anxiety, HF values were lower in the heart‐focused breathing technique session than in the basal session (*p* = 0.0356). The main variations in HRV parameters were recorded for the relative values of the spectral parameters.

**FIGURE 2:**
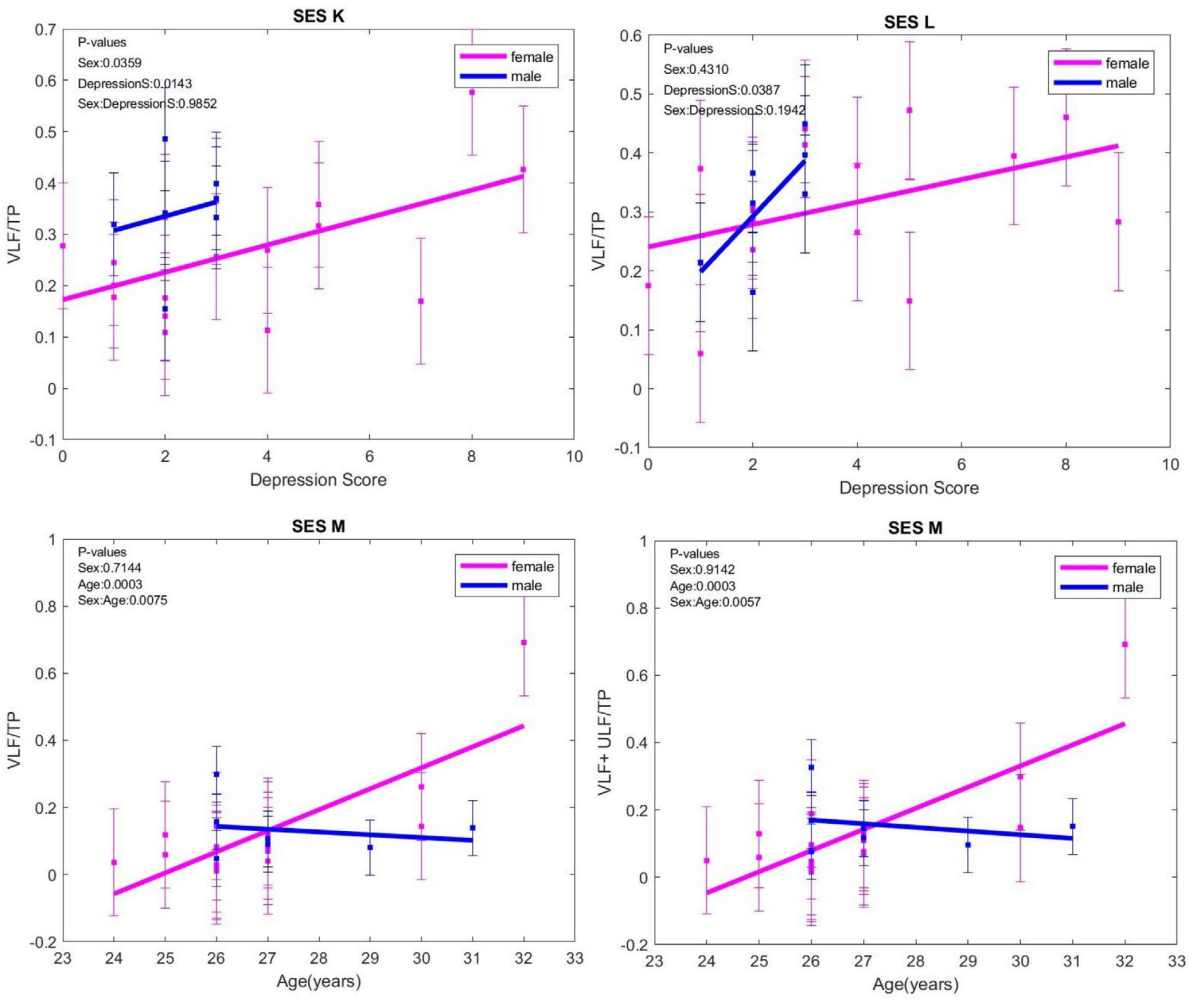
VLF/TP variations at rest (K), when exposed to negative content images (L), and in focused‐breathing (M) sessions. The error bars represent ± one standard deviation (SD) from the mean value: Basal (K), negative images (L), heart‐focused breathing (M), attitude breathing during exposure to negative images (N).

VLF/TP decreased with age in males and increased in females. In this heart‐focused session (*p* = 0.0003), the same evolution with age was observed for VLF+ULF/TP (*p* = 0.0003), which was also noted in this session. In basal and negative image visioning sessions, VLF/TP was directly correlated with the depression score in both sexes (*p* = 0.0183, respectively; *p* = 0.0387). (Figure 2).

VLF/TP and VLF+ULF/TP were lower in all respiratory sessions (M, N) compared with non‐respiratory sessions (K, L), as observed in the general study lot and the subgroup without anxiety. In females with anxiety, VLF/TP was lower in the N session compared to the L session (*p* = 0.0501), a similar pattern to that observed in VLF+ULF/TP for the attitude breathing versus seeing negative images without any breathing control session (*p* = 0.0362) and the N session compared to the K session (*p* = 0.0317).

**FIGURE 3:**
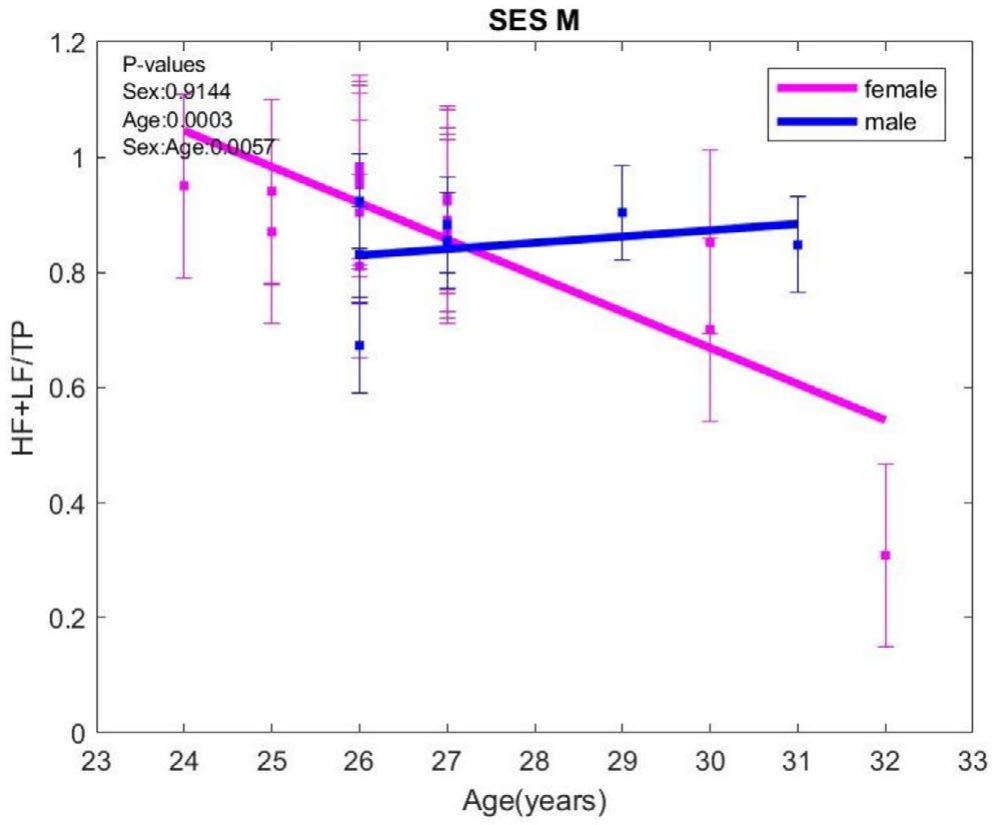
HF+LF/TP evolution with age in heart‐focused breathing (M) session; HF, high frequency; LF, low frequency; TP, total power. The error bars represent ± one standard deviation (SD) from the mean value.

Still, HF+LF/TP, HF+LF/VLF, and HF+LF/VLF+ULF mirrored VLF/TP evolution, being higher in all respiratory sessions compared with the sessions without respiratory control (N vs. K, N vs. L, M vs. K), an effect observed in all the analyzed groups—the general batch, the group without anxiety or depression. In males in the D0–7 group, HF+LF/TP values were higher in N versus K and M versus K situations. HF+LF/TP exhibited an age‐sex‐related evolution for the controlled breathing (M) session, decreasing with age in females and increasing with age in males. (Figure 3).

In females with anxiety, HF+LF/VLF+ULF was higher in the heart‐focused breathing technique (M) compared to the basal state (*p* = 0.0317) and in the attitude breathing technique (N) compared to basal sessions (*p* = 0.0483). HF+LF/VLF was also higher in Heart‐focused breathing sessions compared to basal sessions (*p* = 0.0284).

The nLF and nlf parameters have not changed regardless of anxiety or depression scores. They followed the same evolution: higher in M (heart‐focused breathing technique) compared to K and N (attitude breathing technique) in sessions, without sex‐related differences, in the general population, and in those without anxiety or depression. In all groups, nLF and nlf were higher in L versus K sessions and higher in females.

nhf mirrored nLF and nlf evolution, but in restricted situations, such as N versus K, N versus L, M versus K for the general batch, and M versus K in the A0–7 group, and N versus K and M versus K in the D0–7 group. nHF showed differences only for M versus K sessions. In females with anxiety, nhf was lower in session M than in session K (*p* = 0.0427).

LF/HF recorded the most significant changes noted during the respiratory control sessions compared to those without respiratory control. Thus, LF/HF was higher in session M compared to K, N versus L, and N versus K, without significant differences between the sexes, in both the general batch and the D0–7 group. In those without anxiety and the general batch, LF/HF increased more in males in L versus K sessions. In females with anxiety, LF/HF was higher in N versus K (*p* = 0.0535) sessions.

**FIGURE 4:**
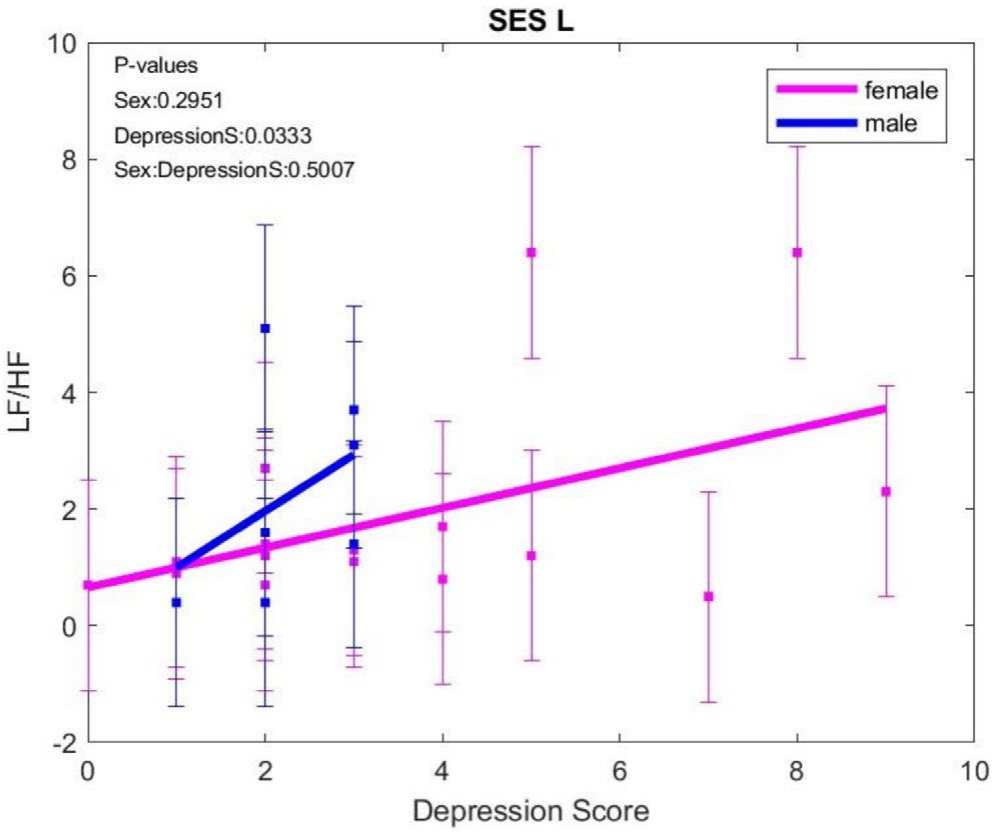
LF/HF variation with age when exposed to negative content images (L) session; HF, high frequency; LF, low frequency. The error bars represent ± one standard deviation (SD) from the mean value.

LF/HF increased with depression score in both sexes, without differences between sexes (Figure 4).

**FIGURE 5:**
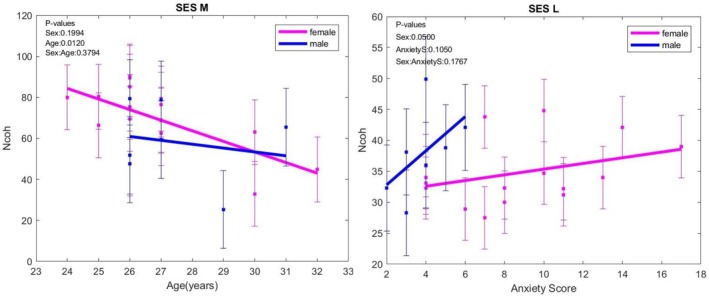
NCoh evolution with age in heart‐focused breathing (M) and during exposure to negative images (L) sessions. The error bars represent ± one standard deviation (SD) from the mean value.

The lowest values of NCoh were observed at rest, sessions K, and the highest value was found in the session using the heart‐focused breathing technique (M), followed by the Attitude‐breathing session. In the M session, NCoh decreased with age in both sexes (*p* = 0.0120). In the L session, NCoh values were higher in males (*p* = 0.0500). NCoh increased in M versus K, N versus K, and N versus L sessions in all analyzed groups, without differences between sexes. In females with anxiety, NCoh was higher in the M versus K session (*p* = 0.0001), and also in the N versus L session (*p* = 0.0009) and the N versus K sessions (*p* = 0.0003). (Figure 5).

### 3.3 Correlations between various HRV parameters (Figure 6)

**FIGURE 6:**
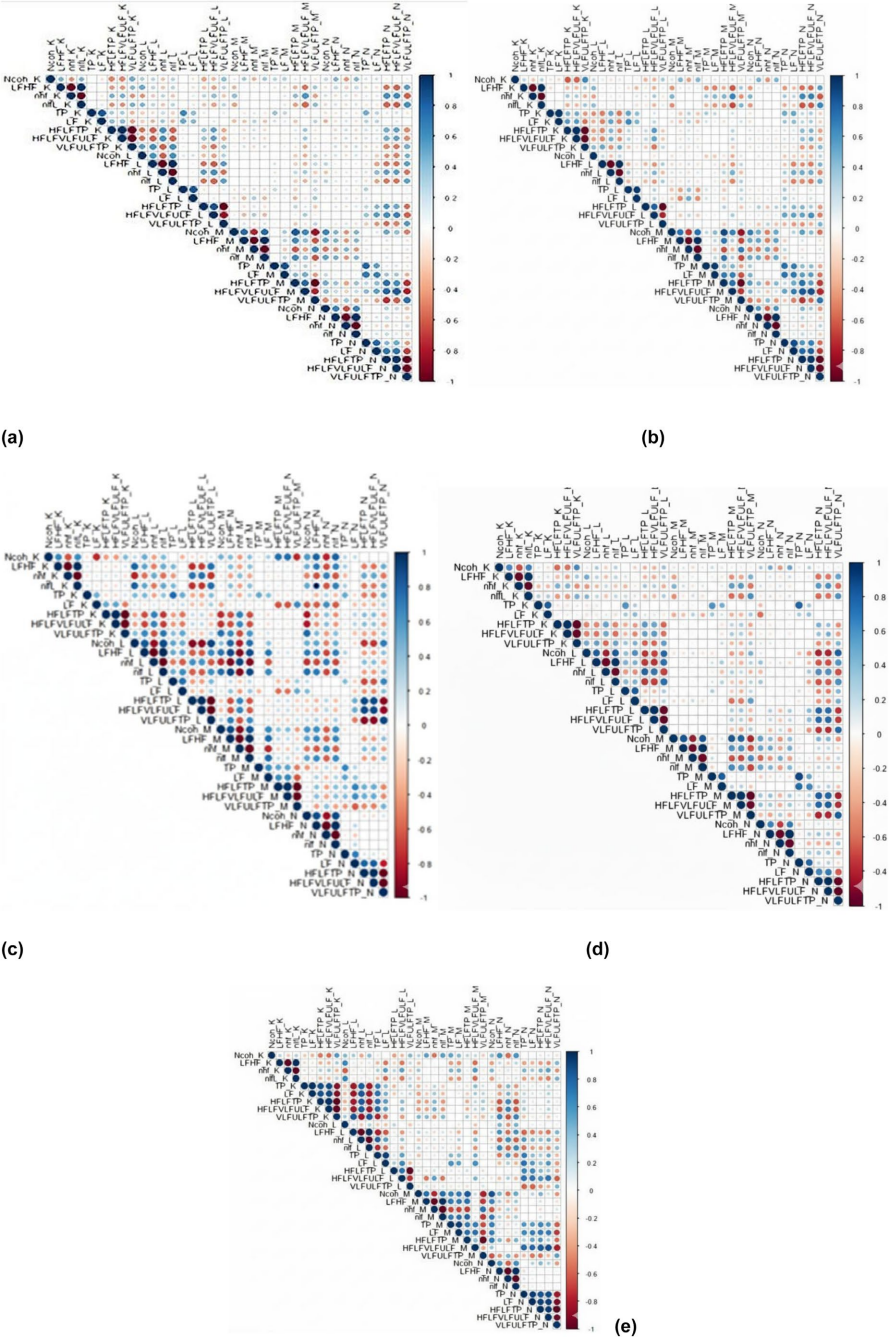
Correlations between the HRV parameters (NCoh, LF/HF, nhf, nlf, TP, LF, HF+LF/TP, HF+LF/VLF+ULF, and VLF+ULF/TP) of different sessions (K, L, M and N), on: (a) the general study cohort (24 subjects), (b) female participants (17 subjects), (c) males participants (7 subjects), (d) subjects without anxiety (15 subjects), (e) subjects with anxiety (9 subjects). Blue colors indicate positive correlations, red colors indicate negative correlations. In contrast, the intensity of the color and the circle size are proportional to the correlation coefficients (larger and intense colored circles indicate stronger correlations); Basal (K); negative images (L); heart‐focused breathing (M); attitude breathing during exposure to negative images (N).

There was no correlation between NCoh of different sessions.

LF/HF is directly correlated with nLF and nLF, except for nLF in session K in males without anxiety, and inversely with nHF and nHF for all sessions.

NCoh correlates with LF/HF in K (*r* = 0.585, *p* = 0.022), L (*r* = 0.794, *p* = 0.000), M (*r* = 0.821, *p* = 0.000), and N (*r* = 0.660, *p* = 0.007) sessions in subjects without anxiety. In general study subjects, we found a correlation between NCoh and LF/HF in sessions K (*r* = 0.464, *p* = 0.002), L (*r* = 0.588, *p* = 0.002), M (*r* = 0.762, *p* < 0.001), and N (*r* = 0.654, *p* = 0.001). In men, we observed a direct correlation between NCoh and LF/HF in the L session (*r* = 0.847, *p* = 0.016), the N session (*r* = 0.857, *p* = 0.014), and an inverse correlation in the M session (*r* = −0.893, *p* = 0.007). In women, LF/HF was correlated with NCoh in sessions M (*r* = 0.728, *p* = 0.001) and N (*r* = 0.577, *p* = 0.015). In females with anxiety, NCoh and LF/HF were correlated only in session M (*r* = 0.745, *p* = 0.021).

In the general group, for the basal session, NCoh correlated inversely with HF+LF/TP (*r* = −0.483, *p* = 0.017) and directly with VLF+ULF/TP (*r* = 0.483, *p* = 0.017). For session M (heart‐focused breathing technique), NCoh correlates inversely with VLF+ULF/TP (*r* = −0.845, *p* = 0.000) and directly with HF+LF/TP (*r* = 0.845, *p* = 0.000), unlike for session K. The same evolution in females, where NCoh is inversely correlated with HF+LF/TP for the basal session (*r* = −0.590, *p* = 0.013) and directly with VLF/TP for session K (*r* = 0.590, *p* = 0.013). For session M it was directly correlated with HF+LF/TP (*r* = 0.890, *p* = 0.000) and inversely with VLF+ULF/TP (*r* = −0.890, *p* = 0.000). In men, NCoh was inversely correlated with HF+LF/TP for session L (*r* = −0.893, *p* = 0.007) and directly correlated with VLF+ULF/TP for session L (*r* = 0.893, *p* < 0.001).

In the subjects without anxiety, NCoh was inversely correlated with HF + LF/TP in session K (*r* = −0.557, *p* = 0.031) and L (*r* = −0.688, *p* = 0.005), and directly correlated for session M (*r* = 0.799, *p* = 0.000) and directly with VLF+ULF/TP in session K (*r* = 0.557, *p* = 0.031) and L (*r* = 0.688, *p* = 0.005), inversely for session M (*r* = −0.799, *p* = 0.000).

In individuals with anxiety, NCoh correlates directly with HF+LF/TP for session M (*r* = 0.833, *p* = 0.005) and inversely with VLF+ULF/TP (*r* = −0.833, *p* = 0.005).

In the general group, TP correlates directly with LF only for sessions K (*r* = 0.847, *p* = 0.000), L (*r* = 0.490, *p* = 0.015), and N (*r* = 0.868, *p* = 0.000). Different evolution occurs between women, where TP correlates directly with LF for session K (*r* = 0.956, *p* = 0.000), L (*r* = 0.941, *p* = 0.000), M (*r* = 0.944, *p* = 0.000), and N (*r* = 0.900, *p* = 0.000), whereas in men, TP does not correlate with LF. The direct correlation between LF and TP is independent of anxiety. Thus, in the group without anxiety, TP directly correlates with LF in session K (*r* = 0.739, *p* = 0.02), L (*r* = 0.804, *p* < 0.0001), M (*r* = 0.796, *p* < 0.0001), and N (*r* = 0.693, *p* = 0.004). In the group with anxiety, TP directly correlates with LF in session K (*r* = 0.983, *p* < 0.001), L (*r* = 0.900, *p* < 0.001), M (*r* = 0.950, *p* < 0.001), and N (*r* = 0.933, *p* < 0.001). In the general group, LF correlates with HF+LF/TP for sessions K (*r* = 0.407, *p* = 0.048), M (*r* = 0.436, *p* = 0.033), and N (*r* = 0.700, *p* = 0.000). LF correlates inversely with VLF+ULF/TP for sessions K (*r* = −0.407, *p* = 0.048), M (*r* = −0.436, *p* = 0.033), and N (*r* = −0.700, *p* = 0.000). In men, LF does not correlate with HF+LF/TP or VLF+ULF/TP, except N session directly with HF+LF/TP (*r* = 0.786, *p* = 0.036), and inversely with VLF+ULF/TP (*r* = −0.786, *p* = 0.036). In women, LF correlates directly with HF+LF/TP in session K (*r* = 0.549, *p* = 0.022), session M (*r* = 0.627, *p* = 0.007), and N (*r* = 0.762, *p* = 0.000) and inversely with VLF+ULF/TP in session K (*r* = −0.549, *p* = 0.022), M (*r* = −0.627, *p* = 0.007), and N (*r* = 0.762, *p* = 0.000). In the group without anxiety, LF correlates directly with HF+LF/TP in session N (*r* = 0.654, *p* = 0.008) and inversely with VLF+ULF/TP in session N (*r* = −0.654, *p* = 0.008). In those with anxiety, LF correlates with HF+LF/TP directly for session K (*r* = 0.917, *p* = 0.001), M (*r* = 0.767, *p* = 0.016), and N (*r* = 0.933, *p* = 0.000), and inversely with VLF+ULF/TP for session K (*r* = −0.917, *p* = 0.001), M (*r* = −0.767, *p* = 0.016), and N (*r* = −0.933, *p* = 0.000).

SDNN was directly correlated with TP in the general batch in all sessions (*r* between 0.630 and 0.886, *p* between 0.000 and 0.001), and in females, also for all sessions (*r* between 0.804 and 0.958, *p* = 0.000). In male subjects, we observed a direct correlation between SDNN and TP in both the M session (*r* = 0.929, *p* = 0.003) and N session (*r* = 0.804, *p* < 0.0001). SDNN is directly correlated with LF in the general batch across all sessions (*r* and *p* between *r* = 0.669, *p* = 0.003, and *r* = 0.889, *p* = 0.000), as well as in females (*r* and *p* values between *r* = 0.664, *p* = 0.003, and *r* = 0.922, *p* = 0.000). In males, SDNN and LF are directly correlated only in the basal session (*r* = 0.964, *p* = 0.000) and in the N session (*r* = 0.669, *p* = 0.003). Irrespective of anxiety score, SDNN was correlated directly with LF in all sessions (*r* and *p* between *r* = 0.539, *p* = 0.038, and *r* = 0.929, *p* = 0.000), and with TP in all sessions (*r* and *p* between *r* = 0.536, *p* = 0.040, and *r* = 0.950, *p* = 0.000) except the N session in those subjects without anxiety.

## 4 DISCUSSION

The heart rate did not show differences between sessions.

The comparative analysis of time domain and spectral parameters between exposure to negative images and basal (L vs. K), heart‐focused breathing and basal (M vs. K), attitude breathing technique versus basal (N vs. K), and attitude breathing technique and exposure to negative images without respiratory intervention (N vs. L) sessions led to the following results.

### 4.1 Time domain parameters

SDNN increased in all sessions, which implies respiratory control compared with the sessions without respiratory control, with no differences between sexes. SDNN decreased in females with anxiety when exposed to negative images compared to baseline.

rMSSD and HF had no variations between sessions, except for a decrease in the heart‐focused breathing session compared to rest in females with anxiety. rMSSD is positively correlated with HF in females with anxiety.

Similarly, literature data showed that higher heart rates are associated with decreased SDNN and rMSSD (Maleczek et al., 2025; You et al., 2024). In a coherence pattern, rMSSD tends to decrease, even if HRV is increased, as a result of an increase in LF (Balaji et al., 2025). The decrease in rMSSD and SDNN does not imply a reduction in HRV or vagal activity (McCraty & Shaffer, 2015). Some authors suggest that the increase in breathing depth causes an increase in the parasympathetic tone, with an increase in SDNN and a decrease in LF and unchanged HF, or an increase in HF and a decrease in LF power (DeBeck et al., 2010; Komori, 2018; Niewinski et al., 2025; Wujtewicz & Owczuk, 2023).

### 4.2 Spectral domain parameters

In our study, TP and LF increased in all sessions with respiratory control compared to those without respiratory control. In females, TP and LF were directly correlated in all sessions, whereas in male participants, no correlation was observed between the two parameters in any session. TP and LF were correlated irrespective of anxiety score. These differences are supported by other literature data that suggest that men and women engage in different autonomic regulation networks during stress, yet these differences are associated with similar emotional stress outcomes (Goldfarb et al., 2019).

In our study, ULF exhibited an inverse evolution, decreasing during the heart‐focused breathing session compared to the rest session. VLF/TP and VLF+ULF/TP decreased in all sessions, which implied respiratory control. Accordingly, the published data found that VLF and ULF analysis relative to TP can reveal links between breathing patterns and autonomic function (Chen et al., 2024). Moreover, in our study, the results showed differences between sexes; in women, TP correlates directly with LF for all sessions, whereas in men, TP does not correlate with LF for any session. Anxiety did not influence TP‐LF correlation because, irrespective of the presence of anxiety, TP directly correlates with LF in all sessions. Regarding the sex differences in these parameters, studies indicate that gender differences in HRV are evident in adolescents and adults, but not in children, likely due to incomplete autonomic maturation (Estévez‐Báez et al., 2018). Meta‐analyses and cohort studies indicate that females generally exhibit higher HF power, lower LF/HF ratios, and reduced sympathetic activity, suggesting greater parasympathetic dominance and potential cardiovascular protection (Estévez‐Báez et al., 2018; Koenig & Thayer, 2016). Proposed mechanisms include prenatal neurodevelopmental influences, sex hormone effects on the brain, and peripheral actions of gonadal hormones (Koenig & Thayer, 2016).

The correlations between LF and HF+LF/TP were different in persons with anxiety or without anxiety. Thus, in subjects without anxiety, LF was directly correlated with HF+LF/TP during attitude breathing. In the anxiety group, LF was directly correlated with HF+LF/TP and inversely correlated with VLF+ULF/TP in basal and both sessions with respiratory intervention.

LF/HF increased in all sessions with respiratory control, without differences between sexes. In subjects without depression, LF/HF increased in both sexes, but more in females.

LF/HF was directly correlated with nlf, nLF, except nLF for basal in male subjects without anxiety, and inversely correlated with nhf and nHF in all sessions.

The literature states that the LF/HF ratio must be interpreted carefully in conjunction with the average HF and LF power values (McCraty & Shaffer, 2015). Accordingly, a high LF/HF ratio can indicate greater sympathetic dominance during effortful tasks, such as physical exercise or stressful situations (McCraty & Shaffer, 2015). However, it may also indicate enhanced parasympathetic activity, as seen during slow, paced breathing when the respiratory frequency shifts into the LF range, promoting a coherent physiological state (McCraty & Shaffer, 2015).

### 4.3 Normalized coherence (NCoh)

NCoh was correlated with LF/HF in all sessions in subjects without anxiety; for those with anxiety, a direct correlation between NCoh and LF/HF was observed only for the heart‐focused breathing session. We observed sex‐related differences in the NCoh‐LF/HF correlation. In men, we found a correlation between NCoh and LF/HF in all sessions except the basal session, and for female subjects, only in the heart‐focused breathing and attitude‐breathing sessions.

The increase in the LF/HF ratio at higher coherence levels is related to a larger increase in LF power, often associated with an increase in afferent vagal traffic (Kromenacker et al., 2018; Quigley et al., 2024). Although LF/HF does not reflect an increase in sympathetic activity, it is associated with a higher coherence (Balaji et al., 2025; Kromenacker et al., 2018; Quigley et al., 2024).

The parameters represented by nLF and nlf exhibited the same evolution as LF/HF, specifically in heart‐focused and attitude breathing, compared to basal breathing. nLF also increased in both sexes when exposed to negative images compared with the basal level, more so in females, without differences between sessions. nHF and nhf had an inverse evolution compared to nLF and nlf, decreasing in sessions involving respiratory control.

A significant increase in NCoh was observed in all sessions and groups in respiratory control over those without respiratory control. The lowest NCoh values were recorded at rest. For the heart‐focused breathing session, NCoh decreased with age in both sexes.

According to some authors, NCoh correlated positively with LF (increases in higher coherence levels) and LF/HF (an indicator of higher vagal activity and higher coherent levels) (Balaji et al., 2025). NCoh is inversely correlated with SDNN, HF, and rMSSD (Balaji et al., 2025).

Respiratory control does not induce changes in heart rate. Still, it does lead to variations in HRV parameters that quantify either primarily sympathetic activity (e.g., VLF and VLF/TP), predominantly parasympathetic activity (rMSSD, HF, nHF, and nhf), both components of the ANS (SDNN, LF, nLF, nlf, and TP), or the sympathetic–parasympathetic balance (LF/HF). Respiratory control appears to promote a better balance between the two ANS components, as indicated by the most consistent change in LF/HF, which increases in all sessions involving respiratory control, with no sex differences. The improvement in sympathetic–parasympathetic balance induced by respiratory control is also supported by the decrease in VLF, VLF+ULF/TP, and VLF/TP in most situations involving respiratory control. SDNN, LF, nLF, and nlf increase during respiratory control sessions. There are sex‐related differences in how respiratory control modulates these parameters: in females, SDNN, LF, and TP correlate across all sessions, whereas in males, the correlation is observed only in sessions involving respiratory control.

LF/HF correlates directly with NCoh across all sessions, with a distinct relationship between LF/HF and NCoh in males versus females during the heart‐focused breathing technique. NCoh was improved by controlled breathing in anxious females, where a direct NCoh–LF/HF correlation was observed only during the heart‐focused breathing and the attitude breathing technique.

Our study has several limitations, including the heterogeneous distribution of subjects according to their depression scores. Additionally, the recordings included a brief training period in Heart‐focused breathing and attitude breathing techniques. Due to the small number of participants in our study, caution should be exercised when interpreting the findings. However, the strengths of our study include the significant variation between the two sexes, as well as the presence of anxiety in the parameters of heart rate variability and the impact on respiratory control. Our study's novel approach was to better delineate the sympathetic/parasympathetic contribution (though not definitively) by introducing the analysis of VLF, VLF/TP, and HF+LF/TP, in addition to those commonly studied in similar research, which were also analyzed.

## 5 CONCLUSION

Heart rates remained stable throughout the sessions; however, patterns related to sex and condition were observed in heart rate variability parameters (HRV). Within depressive females, SDNN and rMSSD values were the lowest. SDNN was correlated with TP and LF in all sessions, both in the general study population and in females. Irrespective of anxiety score, SDNN was correlated with TP and LF in all situations, except the attitude breathing session in those subjects without anxiety.

However, SDNN showed an increase with respiratory interventions. During breathing control, LF increased, as indicated by LF/HF, HF+LF/TP, HF+LF/VLF+ULF, and HF+LF/VLF, whereas VLF/TP and VLF+ULF/TP showed lower ratios in these sessions. nLF and nlf had higher values in respiratory sessions, mostly in males, mirroring the evolution of nHF and nhf.

NCoh was highest during heart‐focused breathing and lowest at rest. There were no differences between sexes when comparing respiratory sessions and sessions without respiratory control; however, there were differences between sessions. NCoh was improved by controlled breathing in anxious females, where direct NCoh–LF/HF correlation was only during the heart‐focused breathing and the attitude breathing technique. TP exhibited correlation with LF in females but not in males. NCoh was inversely correlated with HF+LF/TP in females at rest, but during calm breathing, NCoh became directly associated with HF+LF/TP.

## AUTHOR CONTRIBUTIONS

I.S‐F. was involved in conceptualization, study design, writing, prepared the tables, and data interpretation. I.T. was involved in data collection and data interpretation. I.M. and C.P. were involved in statistical analysis and figures and data interpretation. S.B. was involved in writing and reviewing, and prepared the tables, data interpretation. All authors reviewed the manuscript.

## FUNDING INFORMATION

Universitatea “Lucian Blaga” din Sibiu (ULBS): Ionela Maniu, LBUS‐IRG‐2024; Universitatea de Medicină şi Farmacie “Carol Davila” Bucureşti (UMF Carol Davila): Ilinca Mihaela Savulescu‐Fiedler, Program Publish Not Perish.

## CONFLICT OF INTEREST STATEMENT

This research received no specific grant from any funding agency in the public, commercial, or not‐for‐profit sectors. The authors declare that there is no conflict of interest.

## ETHICS STATEMENT

Ethical approval for this study was obtained from Coltea Clinical Hospital Ethics Committee (approval number 6006/10.04.2024). Written informed consent was obtained for anonymized patient information to be published in this article. Participants were informed about the study procedures, their right to withdraw at any time, and the confidentiality of their data.

## Data Availability

The data supporting this study's findings are available upon reasonable request.
